# Improving the nutritional values of yellow mealworm *Tenebrio molitor* (Coleoptera: Tenebrionidae) larvae as an animal feed ingredient: a review

**DOI:** 10.1186/s40104-023-00945-x

**Published:** 2023-12-03

**Authors:** Linggawastu Syahrulawal, Magnhild Oust Torske, Rumakanta Sapkota, Geir Næss, Prabhat Khanal

**Affiliations:** 1 Animal Science, Production and Welfare Division, Faculty of Biosciences and Aquaculture, Nord University, Skolegata 22, Steinkjer, 7713 Norway; 2 Department of Environmental Science, Faculty of Technical Sciences, Aarhus University, Frederiksborgvej 399, Roskilde, 4000 Denmark

**Keywords:** Alternative protein source, Feed application, Nutritional value, Yellow mealworm

## Abstract

Yellow mealworm larvae (YML; *Tenebrio molitor*) are considered as a valuable insect species for animal feed due to their high nutritional values and ability to grow under different substrates and rearing conditions. Advances in the understanding of entomophagy and animal nutrition over the past decades have propelled research areas toward testing multiple aspects of YML to exploit them better as animal feed sources. This review aims to summarize various approaches that could be exploited to maximize the nutritional values of YML as an animal feed ingredient. In addition, YML has the potential to be used as an antimicrobial or bioactive agent to improve animal health and immune function in production animals. The dynamics of the nutritional profile of YML can be influenced by multiple factors and should be taken into account when attempting to optimize the nutrient contents of YML as an animal feed ingredient. Specifically, the use of novel land-based and aquatic feeding resources, probiotics, and the exploitation of larval gut microbiomes as novel strategies can assist to maximize the nutritional potential of YML. Selection of relevant feed supplies, optimization of ambient conditions, the introduction of novel genetic selection procedures, and implementation of effective post-harvest processing may be required in the future to commercialize mealworm production. Furthermore, the use of appropriate agricultural practices and technological improvements within the mealworm production sector should be aimed at achieving both economic and environmental sustainability. The issues highlighted in this review could pave the way for future approaches to improve the nutritional value of YML.

## Introduction

The global population is expected to reach 10 billion by 2050, with global food demand increasing by 35%–56% between 2010 and 2050 [1]. Animal products contribute up to 70% of the total food demand globally [2], and there will be increased demand for animal-based products in the future. This is primarily due to shifts in food habits toward animal products and increased socioeconomic status, particularly in low-income areas. To meet the higher demands for animal products, the future livestock sector needs to become more productive and environmentally sustainable. Adopting sustainable feeding strategies is necessary since animal feed is a critical factor in achieving environmental and economic sustainability within the livestock sector. In this context, identifying and utilizing innovative and alternative feed resources, such as insects, could be a viable solution. This is because insects can be produced using low-grade byproducts and bioresources resulting in reduced nutritional competition between humans and production animals [3].

Yellow mealworm larvae (YML) have recently been recognized as a novel feed source with the potential for commercial-scale production in the future [4–7]. Mealworms have high protein content with a similar or even better amino acid profile compared to commercially available soybean-based protein sources for livestock that are currently in use [5, 8, 9]. In addition, YML are rich in fat particularly unsaturated fatty acids, which can be considered as a healthy fat source for production animals [10, 11]. Furthermore, YML contain several health-promoting bioactive peptides that are beneficial as animal feed ingredients [12–14]. Mealworms also have several benefits, including the ability to be grown using low-grade organic bioresources or byproducts, higher feed conversion efficiency, the need for less water and land for growth, and the potential to minimize greenhouse gases [15–17]. Thus, YML can serve as a nutritious and sustainable source of animal feed ingredients in the future.

Multiple factors can affect mealworms' nutritional values, and thus, maintaining and even improving the nutritional values of mealworms remains a critical concern in the mealworm production sector. This requires understanding and utilizing knowledge from several aspects, including metamorphosis, dietary habits, digestive physiology, and associated biochemical pathways of yellow mealworms. In addition, genetic selection, environmental factors, and post-harvest processing could play a crucial role in maximizing the nutritional content of mealworm larval biomass.

The goal of this paper was to identify and evaluate various factors important for the improvement of the nutritional values of YML, and for this, a scoping review was conducted using relevant scientific publications and literature. Scientific literature was retrieved using various online databases, including PubMed, Google Scholar, Scopus, and Web of Science, with specific keywords relevant to this review. In particular, the keywords and search terms used to retrieve relevant studies included: “*Tenebrio molitor*” AND one or combinations of more than one of the following search terms: “animal feed”, “nutritional value”, “life cycle”, “larval growth”, “bioactive compounds”, “antimicrobial peptides”, “bioactive peptides”, “fatty acids”, “polysaccharides”, “digestive system”, “digestibility”, “metabolic response”, “feeding substrates”, “probiotics”, “gut microbiome”, “rearing conditions”, “population density”, “genomic selection”, “feed safety”, “processing”, “economic analysis”.

## The life cycle of yellow mealworm

**Fig. 1:**
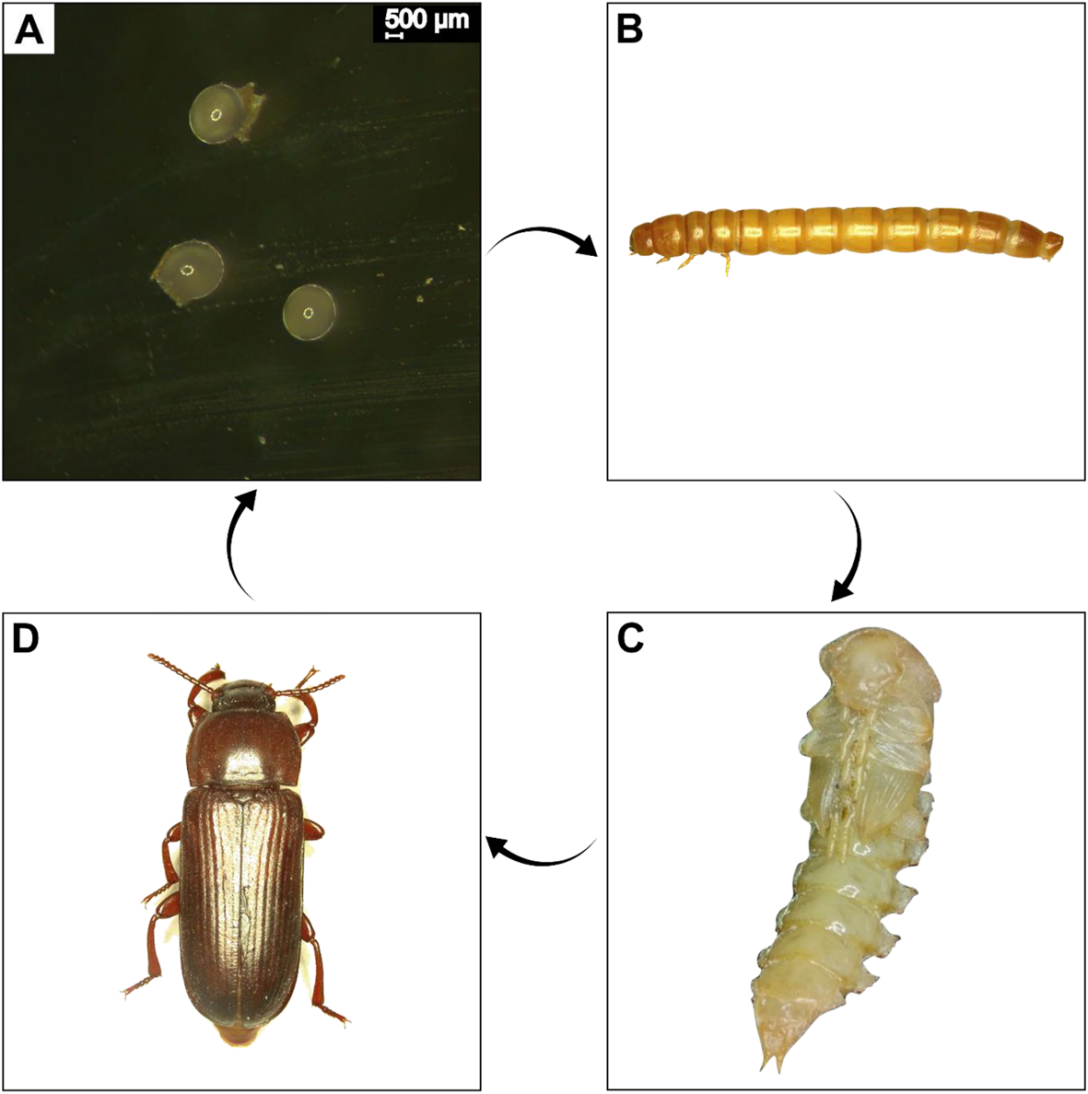
The life cycle of yellow mealworm (*Tenebrio molitor*). **A** Eggs; scale bar 500 μm, **B** Larva, **C** Pupa, **D** Adult

Yellow mealworm is holometabolous with four distinct metamorphic stages: egg, larva, pupa, and adult (Fig. 1). The eggs hatch and become larvae after 3–9 d at 25 °C [18]. The newly hatched larvae are light white and range in length from 0.34 cm to up to 3.16 cm at the 20^th^ instar [19]. Larvae have an elongated, cylindrical form, with mature larvae with an average weight of 0.2 g, being well-sclerotized and having six legs behind the head and two small appendages at the abdomen's ends [20, 21]. The larval stage normally lasts for two to four months, depending on growing temperature and feed availability [5, 19]. The larva eventually stops feeding and forms pupa, during which the insect undergoes metamorphosis and transforms into an adult. The pupal stage last around 5–9 d [18, 22, 23], after which an adult darkling beetle emerges and mates to start the cycle again. The lifespan of an adult ranges between 37–97 d [21]. A recent study has shown that the nutritional value of yellow mealworms differs depending upon their metamorphic stages, with larvae having a higher nutritional value for use as animal feed than pupae and adults [5]. As a result, we will discuss how to improve the nutritional values of mealworms, with an emphasis on the larval stage.

## Nutritional value of yellow mealworm larvae

Yellow mealworm larvae contain 40.2%–63.3% protein on a dry matter (DM) basis, depending upon the types of rearing substrates used to grow them [5, 24–28] (Table 1). For example, larvae fed with wheat bran had a higher (63.3% DM) protein content compared to barley meal (52.7% DM), which could be linked to higher protein in wheat bran substrate [28]. The variations in the protein contents of YML can also be associated with environmental conditions used for larval production. YML grown at a temperature of 25 °C had 8% higher protein content than larvae grown at 15 °C [29]. Moreover, variations in the larval protein may also arise due to inconsistent nitrogen-to-protein conversion factors (Kp) across studies. In general, the protein content of YML in several previous studies was calculated using a Kp of 6.25 [5, 24, 30]. However, it has been suggested that the optimal Kp for YML would be 4.76 based on the amino acid composition to account for non-protein nitrogen sources [31, 32]. Previous studies have found that glutamic acid + glutamate (38–125.3 g/kg DM), aspartic acid + asparagine (5–97.4 g/kg DM), alanine (30.5–100.9 g/kg DM), and leucine (22–109 g/kg DM) are the most abundant type of amino acids in YML (Table 2).

**Table 1:** Nutritional composition of mealworms fed by various substrates in different rearing conditions

**Substrates**	**Crude protein,** **%DM**	**Crude fat,** **%DM**	**Crude** **fiber,** **%DM**	**Ash,** **%DM**	**Strain** **origin**	**Age,** **weeks**	**Temperature/** **humidity**	**References**
Wheat bran	63.3	19.3	–	–	Greece	~ 16	26 °C/50%	[28]
Wheat (by-product)	40.2	31.6	7.6	5.4	Norway	~ 10	–	[5]
Wheat bran	71.2	6.1	10.4	7.5	Poland	–	27 °C/55%	[33]
Wheat bran	45.6	34.5	–	4	Portugal	–	25 °C	[25]
Wheat bran	47.9	26.1	6.8	3.8	Poland	11	28 °C/55%	[26]
Wheat bran	46.4	32.7	4.6	2.9	Korea	–	25 °C/50%	[34]
Wheat bran	68.9	16.9	–	8.1	China	8	28 °C/60%	[35]
Barley (whole grain)	38.9	45.2	6.3	3.5	Poland	–	27 °C/55%	[33]
Oat (whole grain)	66.4	12.1	9.8	6.6	Poland	–	27 °C/55%	[33]
Oat (by-product)	41.7	27.2	7.8	4.9	Norway	~ 10	–	[5]
Rye bran	43.6	30	7.1	3.6	Poland	11	28 °C/55%	[26]
Buckwheat	51.4	34.0	6.7	3.6	Poland	–	27 °C/55%	[33]
Buckwheat	62.7	58.2	–	–	Greece	~ 16	26 °C/50%	[28]
Durum wheat flour	60	41.4	38.8	–	Greece	~ 16	26 °C/50%	[28]
Mushroom spent corn stover	76.2	6.1	–	5.9	China	8	28 °C/60%	[35]
Highly denatured soybean meal	74.3	8.1	–	6.6	China	8	28 °C/60%	[35]
Spirit distillers’ grains	70.1	11.9	–	7.7	China	8	28 °C/60%	[35]
Mix^1^	43.3–44.9	37.8–39.5	11.3–11.5	3.6–3.7	Hungary	8 – 12	20–23 °C	[27]
Mix^2^	40.9	49.5	–	2.81	Italy	–	25 °C/50–60%	[36]
Mix^3^	50.1	34	7	3.9	Italy	–	28 °C/60%	[37]
Mix^4^	44.8	40.4	13.4	4.8	Brazil	12	25 °C/80%	[38]
Vegetable waste^5^	46.3	43.3	8	3	Hungary	10–14	22.5 °C/60%	[39]
Garden waste^6^	42.3	45.2	8.9	3.2	Hungary	10–14	22.5 °C/60%	[39]
Cattle manure^7^	38.9	46.7	9.5	4.8	Hungary	10–14	22.5 °C/60%	[39]

^1^Mixtures of semolina, flour, and oat flakes

^2^Mixtures of 50% bread and 50% cookies

^3^Mixtures olive pomace + wheat middlings (25%:75%)

^4^Mixtures of wheat flour + soybean flour + bocaiuva pulp flour (25%:25%:50%)

﻿^5^Vegetable waste (mixed peels of 10% onion, 25% potato, 25% sweet potato, 30% carrot, and 10% cucumber, with a total water content of 91.4%)

^6^Green garden waste with grass (50% Poaceae species and other common weeds, 25% tree leaves, and 25% branches (populus, salix, pinus, and corylus species)), and a mixture of stone fruits, and other ornamental plant parts, with a water content of 36.2%

^7^55% cattle manure with feces and urine, and 45% cereal straw with a water content of 45.7%

**Table 2:** The amino acids content of yellow mealworm larvae

**Amino acids**	**g/kg DM**
Phenylalanine	12–82
Valine	20–49.2
Threonine	12–75
Tryptophan	4.6–58
Isoleucine	18.7– 32.1
Methionine	5.4–25.2
Histidine	11–35.6
Leucine	22–109
Lysine	12–105.6
Arginine	18–56
Tyrosine	22–67.2
Cysteine	3.9–6.9
Aspartic acid + asparagine	5–97.4
Serine	18.9–66
Glutamic acid + glutamate	38–125.3
Proline	29.9–95.9
Glycine	13–66
Alanine	30.5–100.9

Amino acids contents were obtained from published sources [5, 25, 33, 35, 40]

The fat content of YML is relatively high (~ 22.3%–39.5% DM) compared to other protein sources commonly used in animal feed [5, 24–27] (Table 3). The fatty acids profile of YML showed abundant unsaturated fatty acids (UFA) contents (21.9%–40% of total fatty acids content), whereas the contents of saturated fatty acid (SFA) is lower (22.7%–25.3% of total fatty acids content) [5, 29, 41] (Table 4). Apart from macronutrients, mealworms also contain sufficient minerals to meet the dietary requirement as an animal feed source. The major minerals found in YML studies are sodium (Na) 960–3,644 mg/kg, magnesium (Mg) 2,026–4,100 mg/kg, and potassium (K) 6,440–19,290 mg/kg [5, 25, 34] (Table 5). YML generally have a comparable mineral composition to fish meal and soybean meal (SBM), however, they contain more potassium and magnesium than fish meal and contain higher magnesium and sodium compared to SBM [42]. Despite minerals, larvae were also shown to contain micronutrients like minerals and vitamins [43]. Thus, YML are an excellent source of nutrients, particularly protein and fat, and can potentially substitute traditionally used animal feed ingredients, such as SBM or fish meal, which are widely used feed ingredients.

**Table 3:** The proximate composition of mealworm larvae compared to other protein sources, % (day matter basis)

**Sources**	**Crude protein**	**Crude fat**	**Crude fiber**	**Ash**	**References**
Yellow mealworm larvae	38.9–76.2	6.1–58.2	6.3–11.5	3–8.1	[5, 24–28, 33–39]
Black soldier fly larvae	29.9–48.9	17.1–49	5.2–10.3	4.1–13.2	[44–47]
House fly larvae	42.4–64.6	15.6–25.1	8.7	7.1–9.5	[48–51]
Soybean meal	44.9–49.4	1.4–2.1	5.4–8.6	6.8–10.4	[5, 46]
Chicken feed	18.2–23.2	3.2–8.6	2.9–3.1	4.03–5.2	[39, 52]
Fish meal	42.7–72.0	6.4–16.8	0.6–1	10.2–21.5	[46, 53, 54]

**Table 4:** The fatty acids content of yellow mealworm larvae

**Fatty acids**		**% of total fatty acids**
C12:0	Lauric acid	0.1–0.6
C14:0	Myristic acid	1.7–5
C15:0	Pentadecanoic acid	0.1–0.2
C16:0	Palmitic acid	12.3–19.2
C17:0	Margaric acid	0.03–5.3
C18:0	Stearic acid	0.8–6.3
C20:0	Arachidic acid	0–0.2
Total SFA		22.7–25.3
C16:1	Palmitoleic acid	1.0–2.7
C18:1	n-9Oleic acid (*trans*)	0.1
C18:1	n-9Oleic acid (*cis*)	20.6–54.8
C22:1	n-9Erucic acid	0.8
Total MUFA		22.3–53.2
C18:2	n-6Linoleic acid	12.9–37.9
C18:3	n-3α-Linolenic acid	0.3–6.7
Total PUFA		18.5–46.4
Total UFA		21.9–40
n-6:n-3		2.81–38.6
SFA:UFA		0.3–0.4

Fatty acids contents were obtained from published sources [5, 29, 33, 35, 41]

**Table 5:** The minerals content of yellow mealworm larvae

**Minerals**	**mg/kg**
Sodium (Na)	960–3,644
Magnesium (Mg)	2,026–4,100
Potassium (K)	6,440–19,290
Phosphorus (P)	6,640–14,290
Calcium (Ca)	434.6–2,070
Manganese (Mn)	3.3–13.5
Iron (Fe)	46.6–163
Copper (Cu)	7.8–65.4
Zinc (Zn)	82.3–183.7

Minerals contents were obtained from published sources [5, 25, 33, 34]

The nutritional compositions of YML extend further with the reports pointing out bioactive compounds that can potentially be utilized in improving the health and immune function of production animals when YML are used as animal feed components, as discussed below.

## Health-promoting bioactive compounds of yellow mealworm larvae

In this section, we mainly discuss three major groups of bioactive compounds in YML that could be of particular interest from animal feeding perspectives: bioactive peptides (specific antimicrobial and other bioactive peptides), fatty acids, and polysaccharides.

### Specific antimicrobial peptides (AMPs)

The growing interest in using insects as livestock feed has led to several studies focusing on microbiological hazards and potential health-promoting bioactive compounds in YML. Antimicrobial peptides are compounds that have the potential to kill various pathogens, including bacteria, viruses, and fungi [14]. Due to their broad-spectrum antibacterial action, AMPs have been examined as a prospective treatment for a wide range of infections and modulation of immunomodulatory function in livestock [55]. The antimicrobial activity of AMPs influences the microbial community of the body, including regulating intestinal infections against, for example, *Clostridium* and *Salmonella* spp., and stimulates immune system development [56].

Antimicrobial peptide-type cecropins are the best known α-helical peptides synthesized by insects, including YML, and are one of the most extensively investigated antimicrobial peptides [57]. Both Gram-positive and Gram-negative bacteria are susceptible to cecropins [58]. Another AMP specific to YML is defensins, a proline-rich peptide, which are potent against Gram-negative bacteria like *Escherichia coli,* as well as fungi, yeasts, and protozoa [59]. In YML, AMPs such as attacins could inhibit the production of the key outer membrane proteins of developing Gram-negative bacteria, including *E. coli*, thus disrupting the structure of the cell wall and diminishing the ability of the bacteria to multiply [13, 60]. The majority of insect-derived AMPs are cationic compounds that disrupt the membrane of bacterial cells by generating signaling molecules or transmembrane pores [61]. Moreover, AMPs attach to the anion exchange phospholipids and phosphorus groups of Gram-negative bacteria's lipopolysaccharides (LPS) and the peptidoglycan membrane of Gram-positive microorganisms [62]. This indicates that YML could be used as a source of dietary AMPs, which can act as potential antimicrobial agents, enhancing the animal’s immune response and reducing antibiotics use in livestock.

### Other biofunctional peptides

A wide range of bioactive peptides of YML origins have been discovered, and they may improve the functional properties of animal feed. The di- or tripeptides derived from YML possess antioxidant activity and also trigger angiotensin I-converting enzyme (ACE) inhibitors [12], a class of medicine used for chronic heart failure in animals and human [63]. Using a mouse study, two novel peptides (Leu-Glu and Ala-Lys-Lys-His-Lys-Glu) from YML are found to possess natural hepatoprotective properties, protecting against reactive oxygen species-induced cytotoxicity [64]. It has been reported that YML-derived peptides, e.g., LPDQWDWR and APPDGGFWEWGD, could also be used as natural dipeptidyl peptidase-4 inhibitors to control blood glucose levels [65]. Moreover, anti-inflammatory, antidiabetic, and anti-obesity-related peptides have been identified in YML [66, 67]. Although the literature suggests that YML-derived peptides may have multiple biofunctional properties, further investigations are needed to understand the availability and utilization of YML-derived biologically active peptides for animal feed applications.

### Fatty acids

The growth, development, and survival of YML depend on their fatty acid contents. Lauric acid (LA; C12:0), a medium-chain fatty acid (MCFA) in YML, has shown some antiviral and antibacterial properties [68]. Lauric acid and LA-derived mono-ester glycerol, such as glycerol monolaurate (GML; C_15_H_30_O_4_), are reported to have the greatest antimicrobial activity among all MCFAs [69]. Spranghers et al. [70] observed that fat extracts of prepupae black soldiers fly (BSF) reared with a high amount of C12:0 had an antimicrobial effect against *D-streptococci*. Although the anti-microbial properties of LA content have been well documented, their mode of action against a wide range of pathogens has not been fully understood. In humans, the derivatives of α-linolenic acid, including eicosapentaenoic acid (EPA; C20:5 n-3) and docosahexaenoic acid (DHA; 22:6 n-3), are involved in reducing the risk of cardiovascular diseases [71]. YML are also rich in polyunsaturated fatty acid (PUFA). Studies have also found that n-3 PUFAs have implications as anticarcinogenic [72], antidiabetic [73], immune modulator [74], and brain and visual acuity development in humans [75]. Lawal et al. [41] reported that the inclusion of various levels of seed meals rich in n-3 PUFA, such as flax seed, chia seed, hemp seed, and rapeseed to the diet could improve PUFA contents in YML. This indicates that YML have an endogenous ability to naturally accumulate PUFA from dietary sources. Enriching YML with health-promoting fatty acids with optimal feeding practices in mealworms can enhance both the nutritional and bioactive potential of YML as feed or food ingredients.

### Polysaccharides

Polysaccharides such as chitin and chitosan are important bioactive compounds found in large amounts in YML. Chitin is a long-chain polymer of N-acetyl glucosamine, a glucose derivative, and it is the most significant biopolymer and the second most prevalent compound after cellulose [76]. Chitosan is an N-deacetylated chitin derivative produced by deacetylating certain N-acetyl glucosamine moieties into glucosamine units [77]. In biological systems, chitin and chitosan not only serve as chelating agents but also possess antimicrobial activities against microorganisms [77]. Fish, birds, and mammals cannot synthesize chitin or chitosan, however, chitin and chitosan derived from insects could improve and boost their immune function [76]. For instance, chitin from YML has a positive effect on laying hens as a feed ingredient by increasing blood globulin levels and reducing the albumin/globulin ratio [78]. In birds, higher globulin levels and a lower albumin/globulin ratio imply better disease resistance and immunological function [79]. Therefore, the inclusion of YML in the diets of production animals can improve their immune response and overall health via YML-derived chitin and chitosan.

Several studies have indicated that feeding is a primary driver of the nutrient profile and perhaps also the contents of AMPs in YML. Thus, there is a possibility to maximize the nutritional values and other beneficial compounds of YML by optimizing feeding substrates. However, to further identify and implement specific strategies associated with improving the nutritional value of YML, it is vital to understand their digestive physiology and biochemical pathways involved.

## Digestive physiology and biochemical pathways of yellow mealworm larvae

**Fig. 2:**
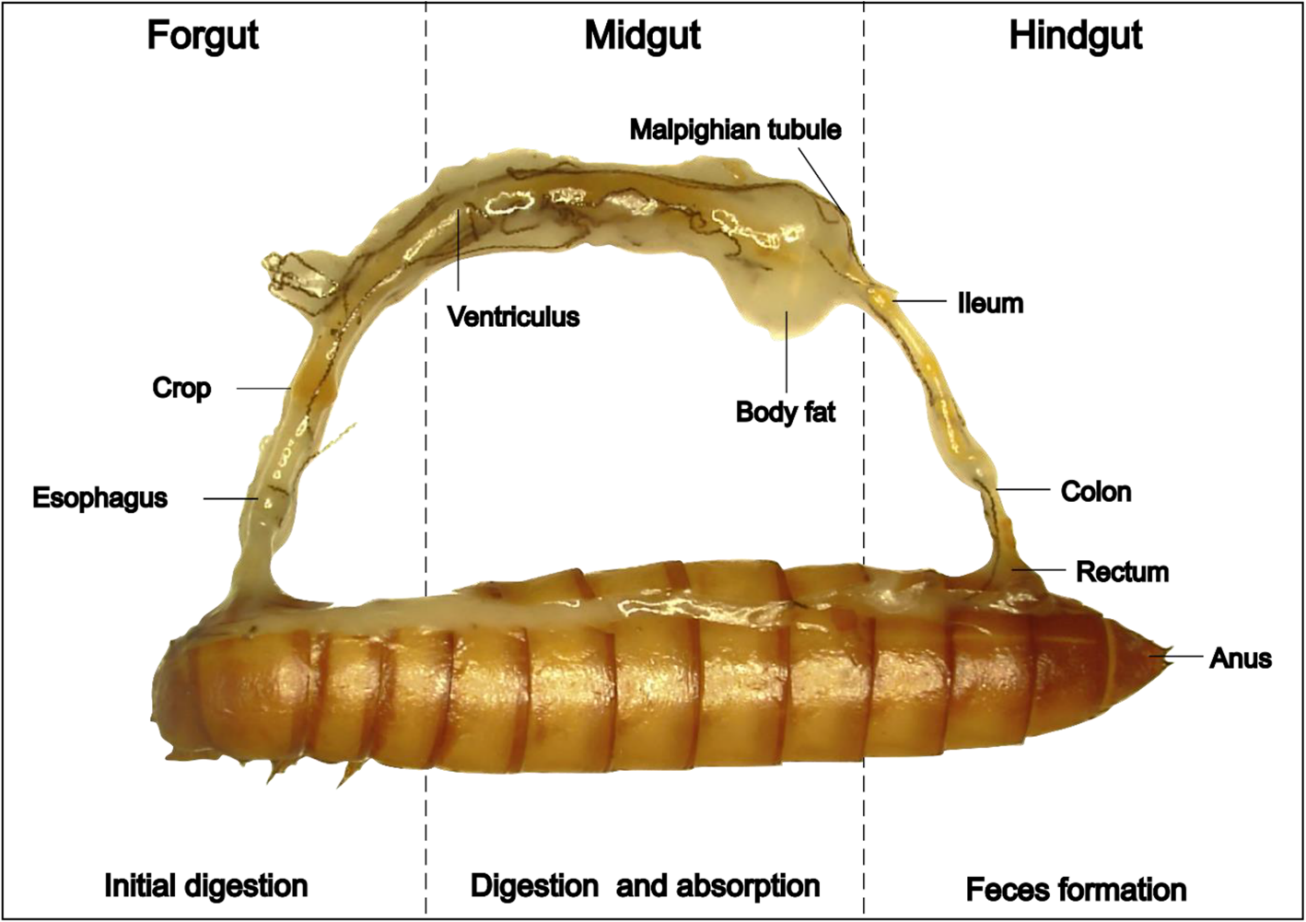
The digestive tract of yellow mealworm larva

The digestive system of YML consists of the alimentary tract, which can be broadly divided into foregut, midgut, and hindgut (Fig. 2). The foregut comprises the mouth, esophagus, crop, and proventriculus, where the crop serves as a storage organ. The proventriculus is the organ involved in crushing small particles in YML, and it controls the entry of feed substrate into the midgut, which is the primary location of feed digestion and nutrient absorption. The midgut of mealworm larvae consists of a simple tube or ventriculus, that is coated by a peritrophic membrane that divides the lumen content into two parts: the endoperitrophic space and the ectoperitrophic region [80]. Transcriptomic analysis has shown that sugar and amino acid uniporters and symporters are expressed in the YML midgut, where the majority of water and nutrient absorption occurs [81]. When the partly digested feed substrate reaches the hindgut of YML, further breakdown of feed takes place by enzymes and microbes, and then nutrients are absorbed [82]. The hindgut of YML consists of the ileum, colon, and rectum. It has structures holding ingested feed and hosting microbes, and the microbes aid in cellulose breakdown producing acetic acid [80]. The digestive structures and enzymes play a crucial role in the digestion of feeds rich in various nutrients.

### Carbohydrates metabolism in yellow mealworm larvae

Mealworms digest polysaccharides such as starch into simple sugars. The YML then absorb simple sugars as an energy source. The larvae produce amylase, α-glucosidases, β-glucosidases, and trehalase from the anterior part of the midgut for carbohydrate digestion [83]. Several structural classes of lectin-like, knottin-like, cereal-type, kunitz-like, γ-purothionin-like, and thaumatin-like compounds are present in cereals and could influence amylase activity in YML [84]. The presence of certain inhibitors, such as toxic proteins in cereals and bean seed extracts, can significantly impede the activity of major digestive enzymes such as α-amylase in the larval gut [85]. This suggests that proper characterization and removal of toxins in feeding substrates can improve the utilization of carbohydrates by mealworm larvae. In addition, application of simple feed processing methods, such as cleaning and gravity separator, can effectively reduce mycotoxin content in wheat [86] prior to feeding YML. The optimal pH range for amylase activity in mealworms is around pH 5.8 [87]. Enzymes such as trehalases that have been partly or entirely isolated from insect guts have optimal pH values between 4.8 and 6.0 [83]. This suggests that gastrointestinal pH plays a vital role in the digestion of carbohydrates in YML. Hence, the manipulation of pH in the feeding substrate of YML could be effective in maximizing the digestibility of feeding substrates and the potential utilization of nutrients by YML. Although studies are limited, mealworms have been shown to be capable of utilizing fermented feed substrates, which can improve the microbial safety of feeding substrates while potentially improving their digestibility [88].

### Protein metabolism in yellow mealworm larvae

Mealworms metabolize protein as a source of energy and structural components for growth and development. Dietary proteins are degraded into their component amino acids and subsequently used in several metabolic processes by YML. In YML, the anterior midgut was responsible for 64% of all proteolytic activity, whereas the posterior midgut contributed 36% [89]. The YML’s midgut contains a crucial enzyme, dipeptidyl peptidase 4 (DPP4), a proline-specific serine peptidase, that can effectively digest proteins in wheat. The DPP4 was stable in the pH range of 5.0–9.0, with an optimum activity at pH 7.9 [90]. Serine endopeptidases, particularly trypsin, and chymotrypsin, are abundant in the posterior midgut of YML and need alkaline pH for their efficient activity [91]. As for carbohydrates, the presence of certain inhibitors, such as toxic proteins in cereals and bean seed extracts, can significantly impede the activity of proteases in the larval gut [85]. This suggests that proper characterization and removal of toxins in feeding substrates can improve the utilization of proteins by mealworms [86]. In addition, feed fermentation using *Lactobacillus plantarum* has been shown to improve protein digestibility in vitro [92], and such approaches could also be applicable for YML to improve the utilization of protein contents in their feeding substrates.

### Fat metabolism in yellow mealworm larvae

Mealworm larval diets include various feeding resources that contain lipids. The midgut of YML releases the digestive enzyme lipase, which breaks down dietary fat into fatty acids and glycerol [93]. Insects do not have bile salts and have developed other strategies to facilitate lipid digestion [94]. In insects, triacylglycerol lipase may generally hydrolyze 2-monoacylglycerol after displacement from the fatty acid into the 1 position, which is favored by the alkaline midgut pH [95]. Gut conditions such as pH affect the digestive process and enzyme activity in YML. The pH may influence the solubility of digested feed substrate components, fat-digesting enzymes, and the population of gut microbes in YML. The pH of the larval gut can vary depending on the insect species and the type of feed substrates being digested, but it generally ranges from 5.2–5.6 to 7.8–8.2 at the posterior part of the insect larval midgut [89]. However, studies focusing on specific mechanisms and processes leading to fat metabolism in YML are limited.

Studies suggest that lipid profiles in YML are associated with fatty acid contents in the feeding substrates. For example, the omega-6 and omega-3 fatty acid contents were increased in YML when fed with dried brewers spent grains (BSG), which are rich in unsaturated fatty acids compared to wheat bran [96]. Fatty acids-rich feeding resources such as seed meals can improve health-promoting PUFA contents in YML [41, 97]. Alpha-linolenic acid (ALA; C18:3) can be transformed into eicosapentaenoic acid (EPA; C20:5 n-3) and docosahexaenoic acid (DHA; C22:6 n-3) by several enzymatic reactions in YML [98]. The same enzymes may also prolong C18:2 (LA; linoleic acid n-6) [99]. Because of substrate competition, the concentrations and ratios of n-3 and n-6 PUFAs control the amount of the end products generated in those enzymatic processes. Thus, YML are plastic to changes in nutritional composition in feeding substrates, particularly fatty acid contents, and it affects the growth and development of larvae as well as their nutrient profile. Such properties of YML larvae could be exploited to improve the nutritional profile of YML by selecting their relevant feeding substrates for various animal feed or human food applications.

## Factors affecting the growth and nutritional values of yellow mealworm larvae

### Feeding substrates

As discussed earlier, YML diets could be a valuable tool to improve the nutritional content in mealworm larval biomass [27, 33, 36, 38, 100]. Numerous feeding substrates have been used for YML production, and an overview of their larval nutritional profile is outlined in Table 1. Studies show that agricultural by-products such as wheat bran, among various substrates, could be an important feeding resource for YML. Wheat bran or wheat by-products are rich in protein, fiber, and soluble carbohydrates and can positively influence the growth and development of mealworms [28]. The protein contents of YML fed with wheat bran ranged between 45.6%–71.2% DM, whereas the fat content was 19.3%–31.6% DM [25, 33]. Mealworms could also be fed various biowaste, including fruits, vegetables, and cereals. However, moisture optimization in such biowaste before feeding to YML may be necessary. The utilization of such biowastes may assist in decreasing organic waste and provide mealworms with a sustainable feeding supply. Even though organic wastes may have lower nutrient concentrations compared to commercial chicken or wheat bran, they may assist in achieving a normal or even higher protein (~ 46% DM) and fat (~ 43% DM) content in YML [39]. In addition, dietary sources containing bread and cookies also appear to supply adequate nutrients to mealworms [36] (Table 1). Most previous mealworm studies have focused on regular or conventionally used feeding resources. Since rearing substrate is a critical component affecting the growth and nutritional value of YML, it is essential to identify, evaluate and utilize novel and alternative feeding resources for mealworm feeding along with traditional bioresources feeding substrates.

Apart from feeding substrates that have already been tested in YML, various novel feeding substrates can be tested in the diets of YML. Marine macroalgae, also known as seaweeds, are rich in carbohydrates, fiber, and minerals [101]. In fact, limited studies have shown that seaweed biomass can be utilized for insect diet, for example, in the feed of black soldier fly (BSF) [102, 103]. However, there is no data regarding the potential of using seaweeds in the diets of YML. The inclusion of seaweeds in the diets of YML could perhaps improve the nutritional profile and the contents of bioactive compounds, as seaweeds are rich in PUFAs, and various polyphenolic compounds [101, 104, 105], however, future studies are needed to confirm this hypothesis. Moreover, it should be kept in mind that using complex carbohydrate sources, e.g., seaweeds [106], in the diet of YML may have some challenges in regard to the digestibility of structural carbohydrates and may interfere with the growth and development of YML. Conversely, YML could digest alternative agricultural and low-quality feeding substrates including distillery by-products [107]. Future studies are needed to evaluate the potential of alternative land-based and aquatic bioresources to incorporate in future mealworm feeding and improve the nutritional profile of YML.

### Use of probiotics

Prebiotics and probiotics in rearing substrates may boost the development of beneficial larval gut microbes and improve the insect's capacity to digest feed and absorb nutrients, positively affecting the overall health status of YML. A previous study showed that using *Pediococcus pentosaceus*, a lactic acid bacterial strain isolated from the gut of YML, as a probiotic can improve YML growth and survival into adulthood [108]. In addition, using *Bacillus subtilis, Bacillus toyonensis,* and *Enterococcus faecalis* as probiotics resulted in substantial enhancements in growth performance, time to pupation, protein, and total SFA contents of the YML [109]. This further decreased the population of *Enterobacteriaceae* by 46% and 99% in the *B. subtilis* and *B. toyonensis* series, respectively, along with lowered coliforms and endospores with all probiotic series in the YML gut [109].

Several microbes in the gut of YML positively impact larval growth, such as by enhancing nutrient absorption and preventing harmful microorganisms. This process includes the release of antimicrobial substances by gut microbes, such as organic compounds and hydrogen peroxide that might hinder the development and survival of dangerous bacteria [110]. In addition, various lactic acid-producing gut bacteria may induce the development of host defensins by gut epithelial cells and inhibit the adherence of pathogens in YML [111, 112]. These studies indicate that gut microbiome could be used as a novel tool to improve the nutrient metabolism and overall growth performance and health status of YML.

### Gut microbiome of yellow mealworm larvae: A novel tool to improve substrate utilization

**Fig. 3:**
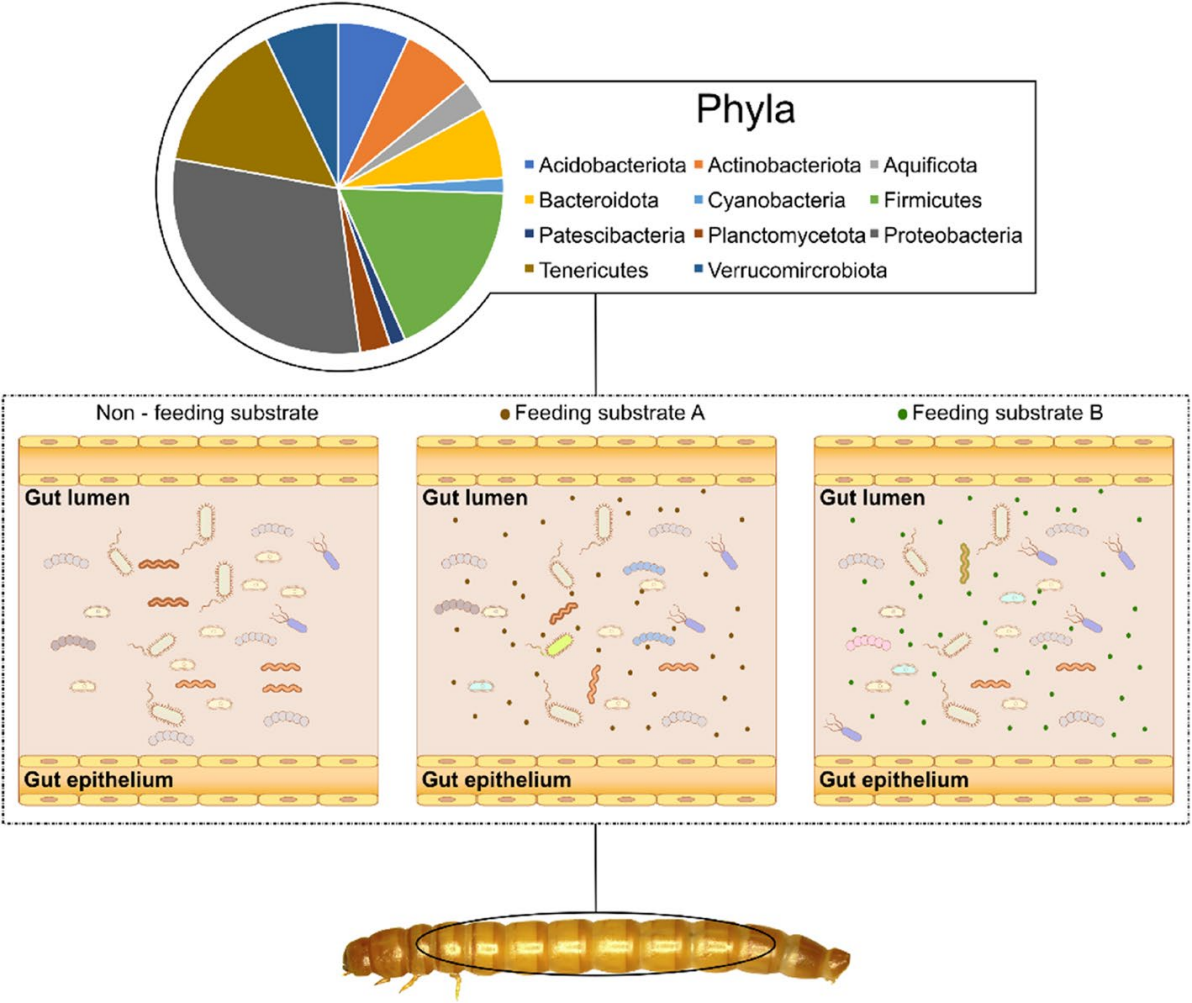
The microbial properties and their response to various feeding substrates (adapted and modified from published literature [5, 115, 117, 118]). The non-feeding substrate column represents the gut nutritional environment where yellow mealworm larvae (YML) are exposed to immediate nutritional challenges, such as fasting. The feeding substrate A column represents an illustration of gut microbial communities of YML generally exposed to cereal-based feeding substrates, such as wheat bran. The feeding substrate B column represents an illustration of gut microbial communities of YML exposed to non-conventional feeding substrates, such as polyethylene and polystyrene wastes

Polyphagous insects, such as mealworms, ingest a variety of feed materials, including plant materials, biowastes, and feces (Table 1). Mealworm larvae can utilize such diverse feed resources with the help of their gut microbiota (Fig. 3). When partially digested feed substrates reach the hindgut of YML, enzymes and microbes break them down before the nutrients are absorbed further into the larval body [82]. The hindgut of YML is adapted for fiber breakdown, by symbiotic microorganisms such as protozoa and bacteria [113, 114]. The gut microbiota can assist the digestion and absorption of complex substances such as cellulose, lignin, and chitin in feed. Interestingly, it has been reported that the gut microbiota of YML can also degrade polymer waste, including polyethylene and polystyrene, that are difficult to digest by other insects or animals [115, 116]. The proportion of YML gut bacterial genera varies among studies. However, the abundance of certain microbial phyla, mainly Proteobacteria, Bacteroides, Firmicutes, and Actinobacteria tend to be consistent, particularly when grain-based bioresources and products are used as rearing substrate [5, 115, 117] (Fig. 3).

Along with a specific function on feed degradation, the gut microbiome of YML is also linked to other physiological tasks, including the regulation of gut pH, the maintenance of a healthy gut, and the production of antimicrobial compounds that defend against harmful microorganisms [119]. The gut microbiota could be used as an additional flexible metabolic tool for host insect species. In whiteflies (*Bemisia tabaci)*, for example, changes in gut microbiota composition have been shown during the adaptation process of switching insects from watermelon to pepper media. Insects grown on pepper for more than two generations showed a significant increase in specific genera, such as *Mycobacterium*, which corresponded to xenobiotic, secondary metabolite degradation pathways, and a substantial rise in insect survival [120]. In addition to the critical role of gut microorganisms in insect growth and survival, they can play an important role in energy and protein metabolism. For example, pyrroloquinoline quinone-dependent alcohol dehydrogenase activity in the commensal bacterium *Acetobacter pomorum* in *Drosophila* was shown to be responsible for the modulation of host insulin/insulin-like growth factor signaling affecting their body size and energy metabolism [121]. Insects feed upon low-grade feed substrates with high carbohydrates and poor protein contents, however gut microbiota of insects plays a significant role in the nitrogen metabolism, promoting their growth, survival, and reproduction. For example, a main gut bacterium, *Candidatus Erwinia dacicola*, isolated from olive fly (*Bactrocera oleae*) was found to supply essential amino acids and help to metabolize urea, improving their egg production [122]. Moreover, gut microbiotas, for example, *Morganella morganii* and *Klebsiella oxytoca*, are found to be important in recycling nitrogenous wastes in the tephritid fruit fly (*Bactrocera dorsalis*) [123]. Specific studies focusing on the role of gut microbiota on the energy and protein metabolism of YML are limited. However, studies from other insect species suggest the potential of manipulating the YML gut microbiome to improve substrate utilization, growth performance, and overall health status.

### Environmental rearing conditions for yellow mealworms

Environmental conditions play a central role in YML production. The mealworm larvae are cold-blooded animals, owing to their reliance on environmental heat for metabolic function. Temperature is a critical environmental factor that can affect the growth and nutritional profile of YML. It has been reported that the optimum temperature for rearing YML is 25–28 °C, whereas the optimum relative humidity is 60%–75% [124]. High energy assimilation efficiencies for YML showed a peak at 23–31 °C [125]. A study showed that the protein and fat content of YML was higher (53% DM) at 37 °C compared to those reared at 31 °C (38% DM) [125]. A temperature changes from 15 to 25 °C could also increase several amino acid compositions, including valine, arginine, and leucine in YML [29]. Unlike protein, larval fat content was higher when rearing at 31 °C (47% DM) compared to 37 °C (30% DM) [125]. Another study showed the highest fat accumulation (30% DM) at 20 °C and the lowest at 15 °C in YML [29]. These studies suggest that environmental parameters, such as temperature, differentially influence the nutritional values of YML, as a higher temperature may promote protein deposition compared to fat.

### Population density

**Fig. 4:**
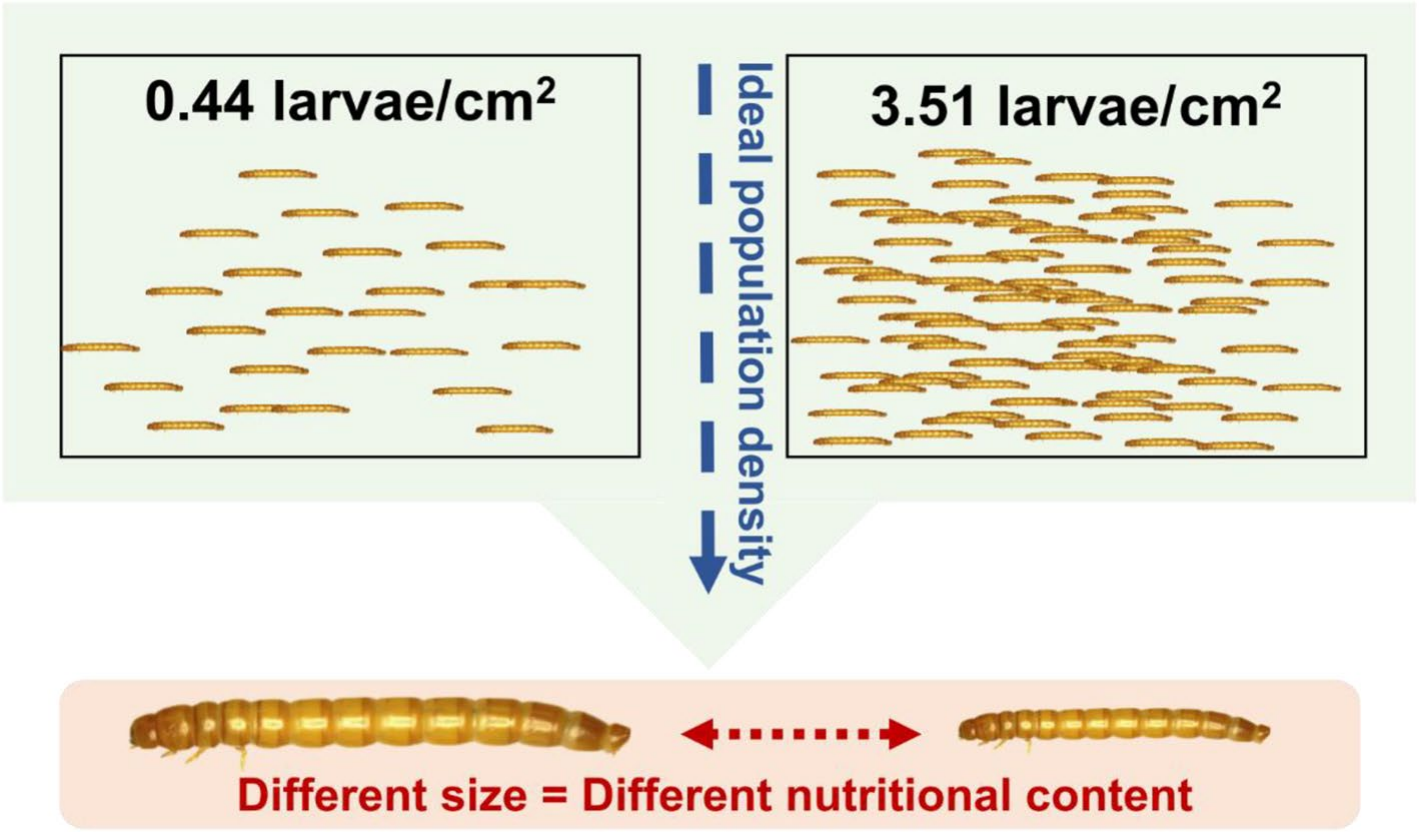
The representative population density and their effect on nutritional compostion of yellow mealworm larva. Adapted and modified from published literature [27, 129]

Population density describes the number of YML individuals per unit of available area (Fig. 4). Population density may have significant effects on the growth and development of YML, as well as on the efficacy and long-term viability of mealworm production [124, 126, 127]. Findings from earlier studies focusing on ideal larval density for YML production are inconsistent. One study reported that the ideal density for larval development was at 0.25 individual/cm^2^ [127], whereas another study suggests that optimal growth of mealworms was achieved with a larval density of 2.5–4 larvae/cm^2^ [128]. Raising the larvae density from 0.44 to 3.51 larvae/cm^2^ could lower the live weight (biomass) gained by 22% [129], suggesting that ~ 0.5 larvae/cm^2^ could be an optimal population density for YML. Ideal larval population density can lead to a greater larval size, potentially accumulating higher amounts of nutrients [27]. In other insect species, for example, black soldier fly (BSF), the larvae produced at a greater density have less fat than those at a lower density [130]. There is a limited number of studies investigating the impacts of population density on nutritional composition in YML. However, population density appears to be an important factor to be considered in the future, particularly in a commercial-scale mealworm production setting.

## Future perspectives

### Genetic selection and improvement

The phenotype of an individual is affected by its genotype, environmental factors, and possible interaction between genotype and environment [131]. Thus, genetic selection and improvement represent a powerful tool to improve the productivity of farm animals. It is widely recognized that genetic selection is an effective approach to improving agricultural productivity and has been practiced in crops and animal improvement dating back to human settlement. Such selection-based productivity improvement approaches can also be applicable to insects, including YML. Progress in insect breeding can be learned from either honeybees or model insects such as the fruit fly (*Drosophila melanogaster*) [132, 133]. A comparative study was conducted to investigate YML-based origin from different parts of the world (Greece, Italy, Germany, Turkey, Spain, and the USA). The study showed that the YML originating from Germany had the highest larval weight at harvest time, whereas the Italian YML performed well in terms of larval survival and growth, biomass production, and feed utilization [100]. This suggests that breeding and selecting a specific strain of YML could be a beneficial strategy to investigate in the future.

**Fig. 5:**
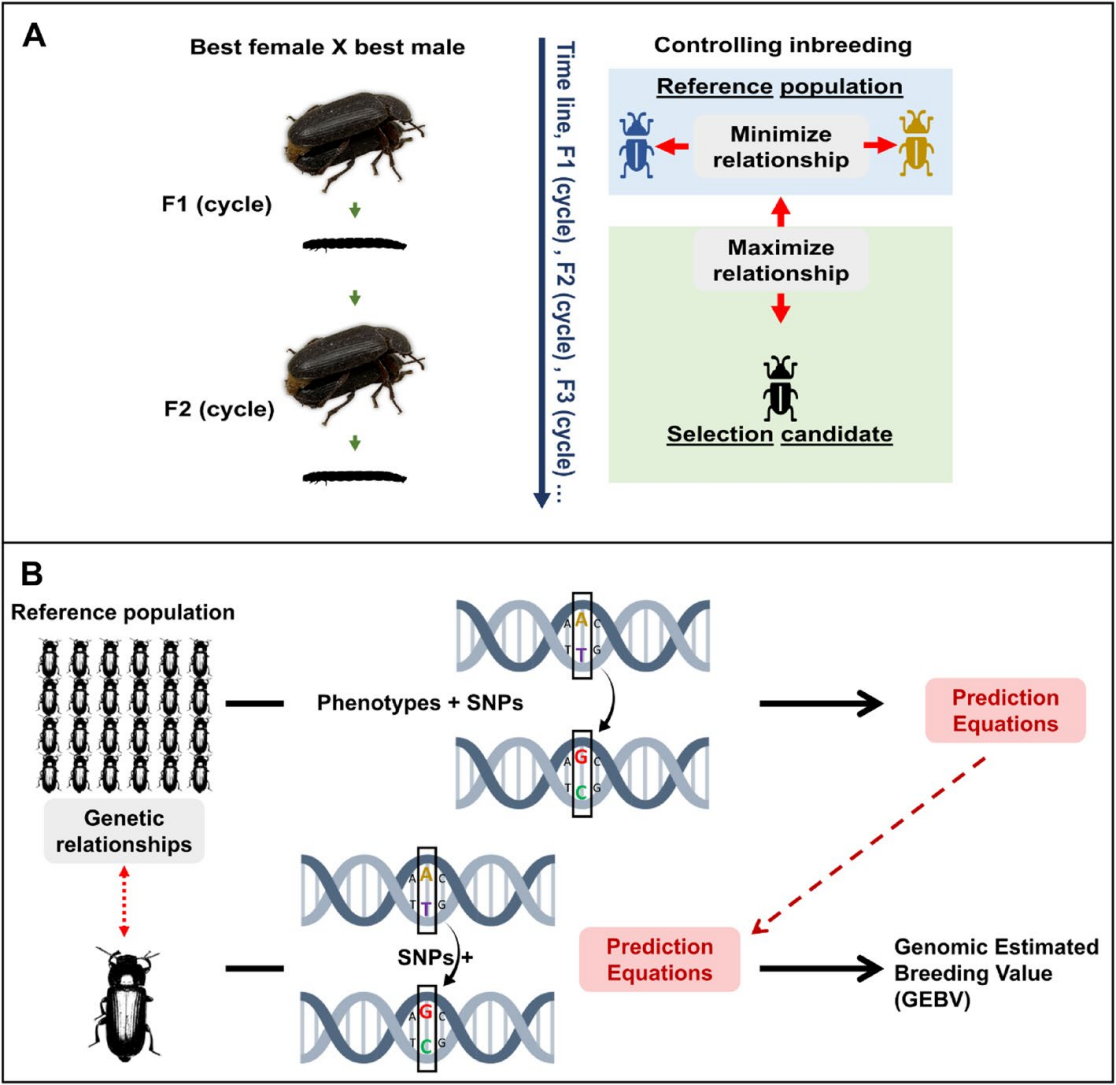
Genetic selection and improvement of yellow mealworm. **A** Breeding program, **B** Genomic selection. SNPs, single nucleotide polymorphisms

Mealworm larval production is still in its early stage, and thus efficient breeding strategies to maintain high-performing larval populations are yet to be implemented (Fig. 5). Given the advantages of the yellow mealworm, which has a short generation interval and produces a high number of offspring, genetic improvement through selection can occur quickly. Hence, it is possible to favor particular genetic traits of YML by using genetic and artificial selection and manipulating growing conditions [134]. Moreover, a high-quality draft genome has recently been revealed to understand the biology of YML further, as ~ 20,305–21,435 gene assemblies have been reported and mapped to allow the development of molecular breeding programs in YML [135, 136]. This further assists in identifying specific genetic markers associated with production qualities [137]. For example, genome-wide association studies (GWAS) can efficiently be used to genotype single nucleotide polymorphisms (SNPs) that are linked to desirable traits [138, 139]. Then, it is possible to predict genomic estimated breeding values (GEBV) of traits of interest in YML as in production animals, such as dairy cattle [140]. Thus, the desirable production traits, such as growth, nutritional values, feed efficiency, etc., of yellow mealworms could be efficiently manipulated using genomic selection as a breeding strategy instead of traditional selective breeding and trait optimization methods.

The theoretical relationship between the inbreeding coefficient and loss of genetic variation across generations varies with population sizes. Smaller populations have greater genetic drift, leading to a loss of genetic diversity due to lower genetic variation and higher inbreeding coefficient than larger populations [131]. Inbreeding leads to homozygosity, which may increase the likelihood that offspring will inherit recessive alleles [141], affecting the population's biological fitness. For example, in livestock breeding, a meta-analysis of thirty years of research on the impact of inbreeding revealed that a 1% increment in pedigree inbreeding was related to a median drop in the phenotypic value of 0.13% of the trait's mean and 0.59% of a trait's standard deviation [142]. To minimize inbreeding and genetic drift in yellow mealworms, genetic variation should be maintained, exploiting both genetic and environmental factors. Therefore, preserving genetic variation is essential as it enables colonies of species to adapt to shifting circumstances and avoid inbreeding, genetic drift, and diseases [131]. Future studies should focus on the genetic improvement and breeding of YML, targeting important production traits of interest, including growth rate, nutritional values, feed efficiency, and fecundity.

### Health and safety of using mealworms and mealworm based-products

Mealworm larvae, like all other food items, must be appropriately processed to ensure the safety of final products. Good agricultural practices (GAPs), including sanitation, are crucial for minimizing the prevalence of hazardous substances [143]. Larvae that have received antibiotics for infectious disease management during production should not be consumed until enough withdrawal period has been applied to ensure no antibiotic residues remain in consumable components [144]. A study also tested mycotoxin deoxynivalenol (DON) degradation on YML and detected that no DON or DON derivates were found in larvae after harvest [145]. This suggests that YML may be able to break down or eliminate DON during production. On the other hand, YML can accumulate heavy metal arsenic in their bodies when their feed is contaminated with arsenic [146]. This suggests that proper evaluation of feed prior to feeding or suitable post-harvesting processing of biomass to remove contaminates is necessary. Nevertheless, raw YML biomass may contain pathogenic bacteria, parasites, and other pollutants that may cause foodborne diseases [147], and suitable treatments are required.

To ensure the feed or food safety of YML-based products, the YML biomass should undergo specific post-harvest processing such as blanching, freezing, and/or drying to reduce microbial loads and other contaminants to safe levels. Freezing is considered the best technique to kill YML and allows immediate preservation and storage of harvested biomass [148, 149]. The decontamination step is preferably performed by microwave or steam blanching to avoid the addition of excess water, which is commonly the case during blanching [147]. After decontamination, larvae are dried, lipids are removed mechanically via pressing, and the resulting press cake is ground into a defatted insect meal [150]. After drying, the growth conditions in YML biomass appear to be unfavorable for critical microbes in terms of food safety, such as *Salmonellae, E. coli, S. aureus, *and* L. monocytogenes*, but dried insects may still carry other opportunistic food-borne pathogens [151], suggesting that further research on multiple post-harvesting processing techniques may be necessary.

Apart from feed applications, there is also a growing interest in using YML as human food. Notably, people with allergies to certain chitin-containing food items, such as crustaceans, crabs, lobster shells, etc., may also be allergic to YML products. A study that evaluated the allergic risk of edible insects cautioned that people allergic to shrimp are more prone to getting an allergy to mealworms and other insects [152]. Chitin can be degraded into smaller pieces by enzymes known as chitinases, which are present in plants, fungi, and animals [76]. When chitinases interact with the immune response of people sensitive to them, chitinases may induce the production of histamine, which can result in various symptoms, including itching, hives, and respiratory issues [153]. However, YML is generally a safe and highly nutritious feeding ingredient when proper evaluation of rearing substrates and appropriate post-harvesting processing techniques are carried out.

### Future farming considerations

In recent years, YML have gained great interest as a reliable and environmentally friendly nutrient source for animal feeding and human consumption. According to Meticulous Research 2023, the global edible-insects market will reach up to USD 16.39 billion by 2032 [154]. The mealworm business is widely expanding, and this brings several factors to consider in the future. Yellow mealworms are one of the more than 2,000 species of insects that have the potential for feed or food application [155]. YML can be used as feed for swine [16, 156], chicken [9, 157], and fish [158]. However, it may be currently difficult to achieve economic sustainability in the insect production sector with low- or medium-scale operations [159]. This is mainly due to the fact that insect-based feed can be more expensive than conventional feed sources [160]. Despite limited reports on the economic aspects of the production of YML species, feed, labor costs, processing methods targeting specific products, and the level of mechanization appear to be key operational costs [161]. Thus, the future insect production sector should focus on upscaling production and commercialization, targeting both economic and environmental sustainability to ensure desirable economic growth while fulfilling demands from animal feed industries.

To provide a nutritious and sustainable protein supply for the future, several factors must be considered, including new potential feeding substrates, legislation, consumer acceptability, and technology. As the mealworm industry expands, this will likely become governed by regional, national, and international regulations. Producers must be aware of and adhere to these regulations to assure the safety and nutritional value of their products. Farmers and business owners in this industry must concentrate on marketing and raising awareness about the beneficial properties of mealworms. In addition, the development and implementation of novel technologies will play a vital role in the future development of the mealworms industry by reducing the cost of production and minimizing any unfavorable environmental footprints.

## Conclusions

The nutritional characteristics of YML for animal feed applications are found to be comparable or even better compared to traditionally used protein sources in animal feed. Various measures and strategies can be implemented to further improve the nutritional value of YML. Given that rearing substrate plays a crucial role in optimizing larval growth and nutritional values, various land-based and aquatic alternative bioresources and byproducts can be evaluated in the future to evaluate the production potential of YML. In the future, the animal feed sector could exploit various antimicrobial and bioactive compounds in YML to improve the health and performance of animals. The use of probiotics has the potential to further enhance the nutritional characteristics of YML. The YML gut microbiome may serve as a useful tool to improve the utilization of diverse feeding substrates. However, the role of gut microbiota on specific nutrient metabolism is yet to be evaluated. Along with dietary factors, environmental parameters play a central role in the growth and development of YML and can differentially affect nutrient assimilation in larval biomass. In addition, maintenance of ideal population density can be an important strategy to optimize larval growth and development and scale up future mealworm production. Further research is needed to fully understand the potential of using conventional or molecular breeding techniques to favor desirable traits and minimize genetic drifts. In addition, various cost-effective post-harvest processing techniques are needed to effectively avoid harmful feed contaminants and microbes. Both economic and environmental sustainability should be considered while exploiting technological advances to commercialize the mealworm production sector in the future.

## Data Availability

Not applicable.

## Abbreviations

ALA: Alpha-linolenic acid
AMPs: Antimicrobial peptides
BSF: Black soldier fly
BSG: Brewers spent grains
DHA: Docosahexaenoic acid
DM: Dry matter
DON: Deoxynivalenol
EPA: Eicosapentaenoic acid
GAPs: Good agricultural practices
GML: Glycerol monolaurate
Kp: Conversion factors
LA: Lauric acid
LPS: Lipopolysaccharides
MCFA: Medium-chain fatty acid
PUFA: Polyunsaturated fatty acid
SBM: Soybean meal
SFA: Saturated fatty acid
SNPs: Single nucleotide polymorphisms
UFA: Unsaturated fatty acids
YML: Yellow mealworm larvae
